# Agrin para-secreted by PDGF-activated human hepatic stellate cells promotes hepatocarcinogenesis *in vitro* and *in vivo*

**DOI:** 10.18632/oncotarget.22186

**Published:** 2017-10-31

**Authors:** Xing Lv, Cheng Fang, Ruozhe Yin, Bowei Qiao, Runze Shang, Jianlin Wang, Wenjie Song, Yong He, Yong Chen

**Affiliations:** ^1^ Department of Hepatobiliary Surgery, Xijing Hospital, Fourth Military Medical University, Xi'an 710032, P.R. China

**Keywords:** hepatocellular carcinoma, agrin, PDGF, sorafenib

## Abstract

Evaluating the process and mechanism of fibrogenesis is essential in hepatocellular carcinoma (HCC), especially in hepatocyte transformation and oncogenic signaling. We evaluated the oncogenic role of agrin secreted by platelet-derived growth factor (PDGF)-induced hepatic stellate cell (HSC) in HCC. Cells were co-cultured to investigate the effect of activated HSC on hepatocytes. Liquid chromatography and protein profiling analysis were used to search the distinct proteins secreted in HSC supernatant. Sprague Dawley rats with Diethylnitrosamine (DEN)-induced HCC were used to simulate human liver cancer and sorafenib was administered to investigate its effect on hepatocarcinogenesis. A paired “two-tailed” Student *t*-test and chi-square tests was used for statistical analysis. PDGF acted as an activator of the HSC and sorafenib inhibits the activation by blocking the combination of PDGF and PDGF receptor. The supernatant of activated HSCs promoted the proliferation, metastasis, and invasion of HL-7702 and SMMC-7721, as well as epithelial-mesenchymal transition (EMT). Agrin found in the HSC supernatant showed the same effect on SMMC-7721 as to the supernatant of activated LX-2. Furthermore, downregulation of agrin by siRNA could decrease the proliferation, metastasis, and invasion of SMMC-7721, and promote MET. Sorafenib prevented DEN-induced hepatocarcinogenesis and could alleviate the liver inflammation and fibrosis. Sorafenib could improve the liver function of Sprague Dawley rats by decreasing the serum levels of ALT and AST. These results demonstrate thatPDGF is an effective activator of HSC and sorafenib could inhibit the activation. *In vivo* experiment suggested sorafenib could alleviate the hepatocarcinogenesis mediated through agrin secretion and could be potential candidate for treatment of cirrhosis.

## INTRODUCTION

The development of cirrhosis and progression to HCC is complex process involving a combination of etiologies. The main risk factor for developing HCC is liver cirrhosis [1, 2]. HCC occurs at a rate of 1% to 4% per year after cirrhosis is established, underlying approximately 80%-90% of cases worldwide [3, 4]. The prevalence rate is 492 cases per million in southern China [5]. As cirrhosis is a silent disease, the prognosis of its progression to HCC is very poor, which is often diagnosed at the later stage when the patient's condition nears death, with a survival time of not more than 1 year [6]. However, with early diagnosis and advanced treatments, the survival time has been prolonged in patients with HCC. The crucial characteristic of cirrhosis progression to HCC is the activation of hepatic stellate cells (HSCs), as activated HSCs increase the production of cytokines, growth factors, and products of oxidative stress, most of which have been shown to affect hepatocyte proliferation by causing liver fibrosis, mitogenesis, and pro-oncogenesis leading to tumor growth and progression, indicating that wound-healing may have a significant impact on HCC development [7, 8]. Among the mitogenic pathways of HSCs, the signaling by the beta platelet-derived growth factor receptor (β-PDGFR) is the most potent [9]. On activation of HSCs by PDGF stimulation, proteoglycans released into the portal circulation are known to exert oncogenic properties. One such proteoglycan is agrin, which is deposited on the walls of HCC tumoral blood vessels. It is studied that the proteoglycan, including agrin, have the potential to bind and modulate the function of growth factors, such as fibroblast growth factor, transforming growth factor-β (TGF-β), vascular endothelial growth factor (VEGF), and PDGF, and numerous cytokines and chemokines [10, 11]. With the growing evidence on the oncogenic role of agrin in regulating focal adhesion integrity and its mechanistic role in tumor microenvironment during hepatocarcinogenesis [12–14], it is plausible that agrin may have a significant role in providing oncogenic microenvironment to the PDGF-stimulated HSCs and HCC development in liver cirrhosis. However, the underlying sequence of this mechanism remains to be explored. For this purpose, we aimed to study the role of agrin in HCC development mediated by PDGF-activated HSCs *in vitro* and *in vivo*. For *in vitro* PDGF receptor expression study, we used a VEGF inhibitor sorafenib with additional PDGF-inhibiting properties, which is one of the drugs approved for the treatment of advanced, non-resectable HCC [15].

## RESULTS

### Activation of HSCs by PDGF and inhibition by sorafenib

**Figure 1:**
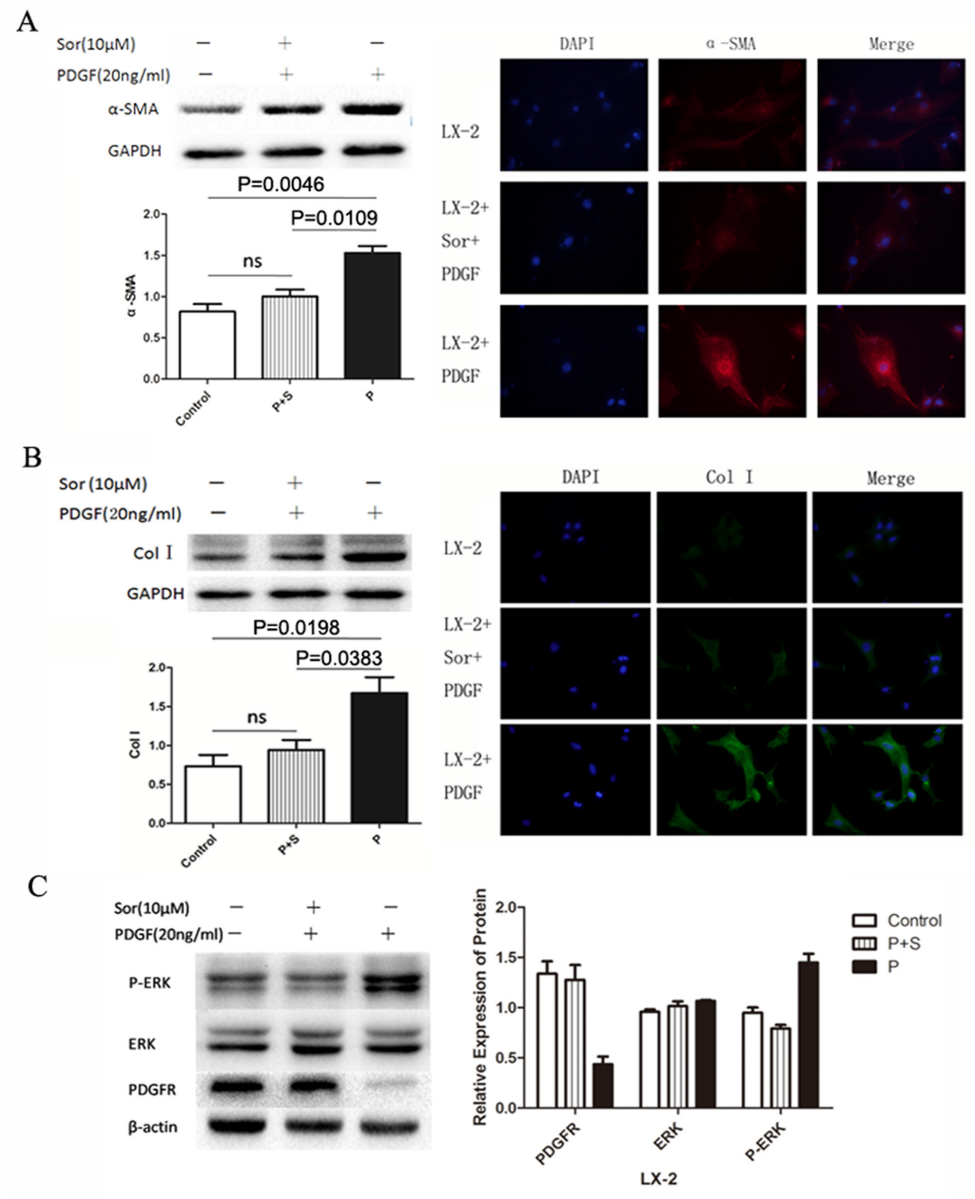
Activation of the LX-2 cell line using PDGF with or without Sorafenib PDGF alone or in combination with sorafenib was used to treat LX-2 cells. Stimulation of the PDGFR signaling pathway was detected by measuring levels of the downstream proteins SMA **(A)**, Col-I **(B)**, and ERK **(C)**. Immunofluorescence staining and western blotting showed that the levels of SMA and col-I increased significantly in the PDGF only group (P), whereas no increase was detected between the control and PDGF + sorafenib (P+S) groups (A and B). Western blotting showed that while PDGF stimulation increased phosphorylation levels of ERK in the PDGF alone (P) group, this increase was inhibited by sorafenib in the combination group (P + S).

To investigate whether PDGF could activate the HSC directly, we stimulated LX-2 with 20 ng/ml PDGF, with or without preconditioned by 10 μM sorafenib. Alpha-SMA and collagen I (col-I), secreted by activated HSCs, were chosen as markers. The expression of α-SMA and col-I increased significantly after the stimulation with PDGF, which showed no change under the pre-treatment with sorafenib (Figure 1A and 1B), indicating that PDGF-activated LX-2 and sorafenib could effectively inhibit the activation. The expression of the markers was further confirmed by immunofluorescence. Furthermore, the total level of ERK protein was not significantly different between sorafenib-treated and untreated cells with PDGF stimulation. However, sorafenib could inhibit the phosphorylation of ERK (Figure 1C).

### Co-culture with activated LX-2 promoted the proliferation, metastasis, and invasion of HL-7702 and SMMC-7721

**Figure 2:**
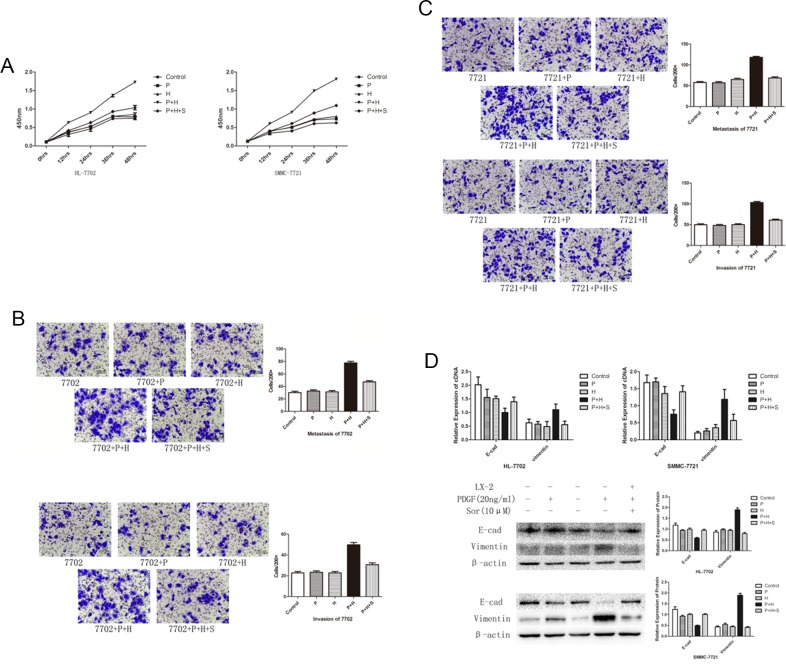
EMT and cell migration in SMC-7721 and HL-7702 cell lines treated with PDGF or sorafenib or activated LX-2 supernatant either alone or in combination **(A)** Effect of different treatments on cell proliferation rates in HL-7702 and SMC-7721 cell lines. A significant increase was detected in cell proliferation rates in both cell lines treated with PDGF-activated LX-2 supernatant (P + H). This increase was inhibited by the addition of sorafenib in both cell lines (P + H + S). **(B and C)** Transwell migration assay results indicated that the rate of cell migration increased in both cell lines treated with PDGF-activated LX-2 supernatant (P + H). This increase was inhibited by the addition of sorafenib (P + H + S). **(D)** Western blot analysis showed that the treatment with activated LX-2 supernatant (LX-2) decreased the levels of E-cadherin and increased level of vimentin. The addition of sorafenib (Sor) inhibited this change in protein expression. Importantly, the use of PDGF alone had no effect on these protein levels in either cell line.

To investigate the effect of activated HSCs, we co-cultured HL-7702 cell line and SMMC-7721 with LX-2 cells. Compared with the control group, the proliferation of HL-7702 and SMMC-7721 increased significantly in the presence of PDGF-activated LX-2 and the cells in the control group remained unchanged (Figure 2A). The transwell assay demonstrated that the PDGF-activated LX-2 could promote the migration and invasion ability of HL-7702 and SMMC-7721 (Figure 2B and 2C). Furthermore, the expression of E-cadherin decreased and vimentin increased significantly (Figure 2D), which indicated that EMT occurred through co-culture with PDGF-activated LX-2. The proliferation capability, migration, and invasion ability and EMT could be alleviated after the pre-treatment of LX-2 with sorafenib.

### Agrin secreted by activated LX-2 induces EMT of SMMC-7721

**Figure 3:**
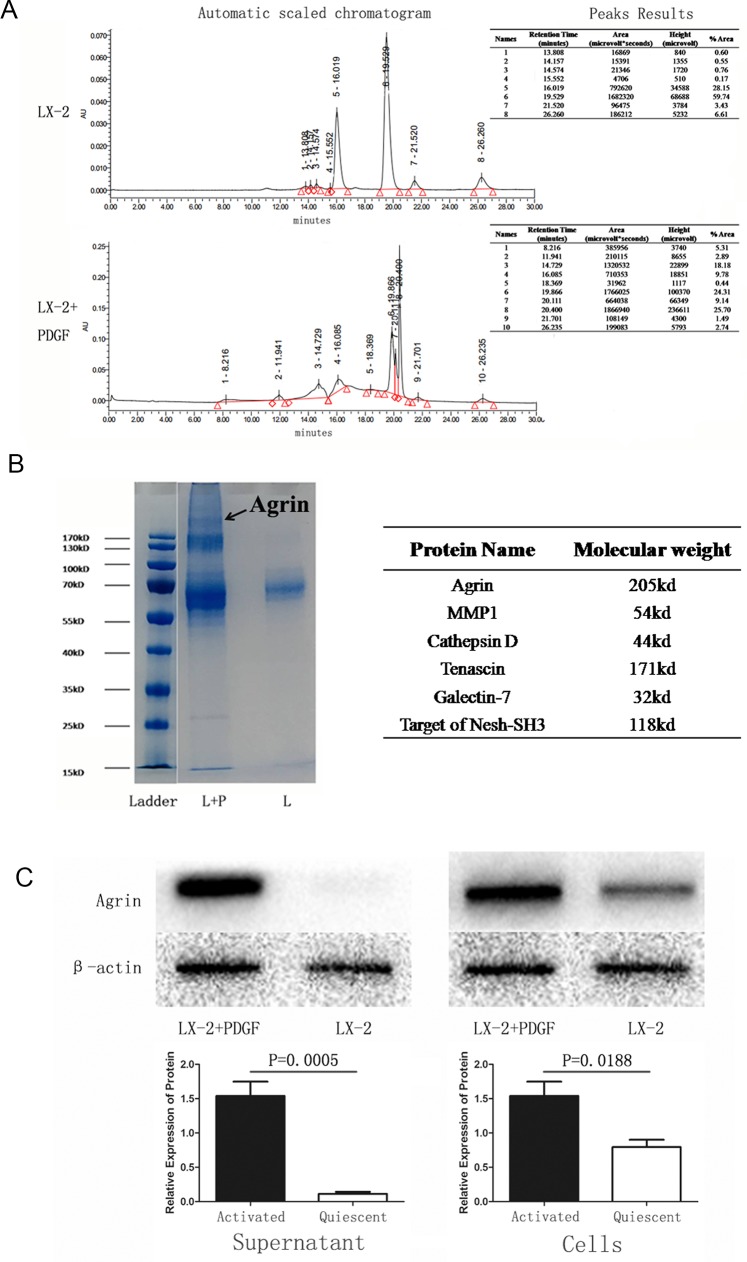
Identification and detection of proteins in the supernatant of quiescent versus PDGF-activated LX-2 cells **(A)** Mass spectrometry analysis of supernatant samples of quiescent versus activated LX-2 cells showing differences in peptides detected. **(B)** Coomassie-stained SDS-PAGE showing the different proteins detected in the supernatant of activated LX-2 cells. A band at MW 200 kDa was seen in the supernatant from activated LX-2 cells, but it was absent in the supernatant from quiescent LX-2 cells. **(C)** Western blotting confirmed that the 200-kDa band comprised agrin (MW = 205 kDa), which was detected only in the supernatant from activated LX-2 cells.

Because PDGF is an activator of LX-2 cells, EMT occurred when hepatocytes were co-cultured with activated LX-2. To study if there were any distinct proteins triggered in the activated LX-2 and quiescent LX-2 stages, we performed liquid chromatography. The chromatograms of the 2 different supernatants are represented in Figure 3A. Gel electrophoresis and coomassie blue staining also showed the difference in fraction (Figure 3B). A protein profiling analysis revealed that agrin was the most important protein triggered in activated LX-2 cells. To further confirm that agrin was secreted by activated LX-2 cells, we performed western blot with both cell protein and supernatant protein collected from activated and quiescent LX-2 cells. The result demonstrated that the secretion of agrin increased by 16-fold in the supernatant protein of activated LX-2 cells (Figure 3C).

### Characterization of functional role of agrin

**Figure 4:**
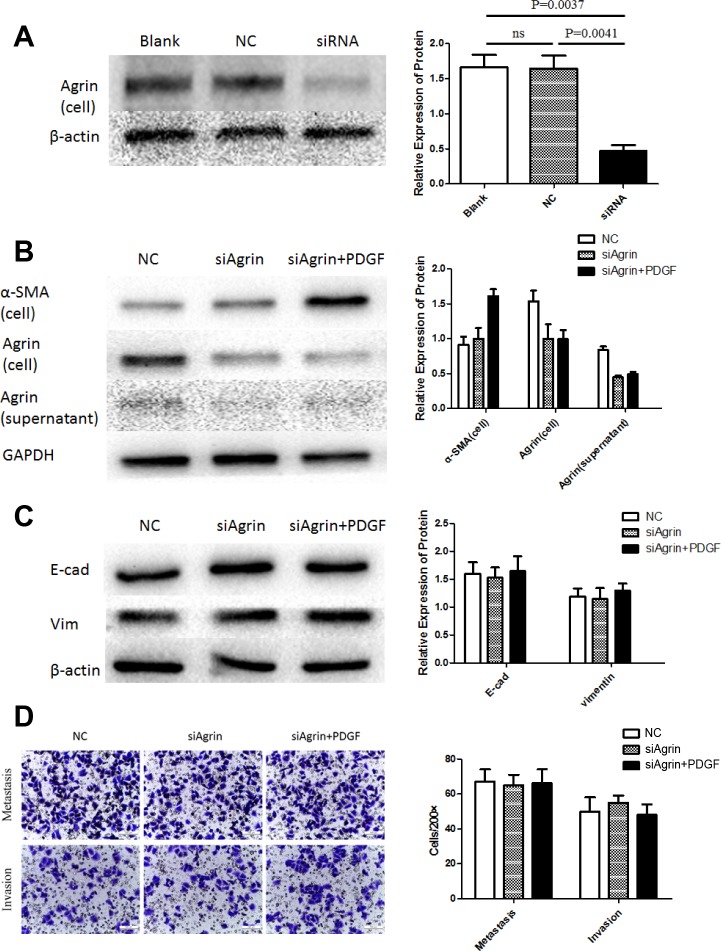
Agrin knockdown and its effect on migration and EMT in LX2 cells **(A)** The effect of siRNA transfection on agrin expression. Agrin protein levels were downregulated in cells transfected with siRNA versus controls. **(B)** Agrin levels in the cell lysates and supernatant were similar between PDGF treated and non-treated cells. **(C)** No difference in the expression of EMT markers (E-cad and Vimentin). **(D)** transwell migration assays showing no difference in the metastatic and invasive ability of the cells.

To characterize the functional role of agrin in HSC and HCC cell lines, agrin was also depleted in the HSC cell line LX-2 by transfecting with siRNA pool, by which effective agrin knockdown was achieved (Figure 4A). In agrin-depleted cells, the expression of agrin was not significantly different between PDGF-treated and untreated cells (Figure 4B). There was significant difference of cell protein (α-SMA) between the control group and the agrin-depleted groups (Figure 4B). However, the expression of agrin in culture supernatant kept on the same level in the agrin-depleted cells, because the level of agrin has been on a very low level in quiescent LX-2 supernatant, and the depletion cannot decrease the secretion of agrin further. As a result, when SMMC-7721 co-cultured with both quiescent and agrin-depleted LX-2, the transwell assay did not demonstrate any difference on the migration and invasion ability (Figure 4D). There were surely no significant differences on the expression of E-cadherin and vimentin as a matter of course (Figure 4C).

**Figure 5:**
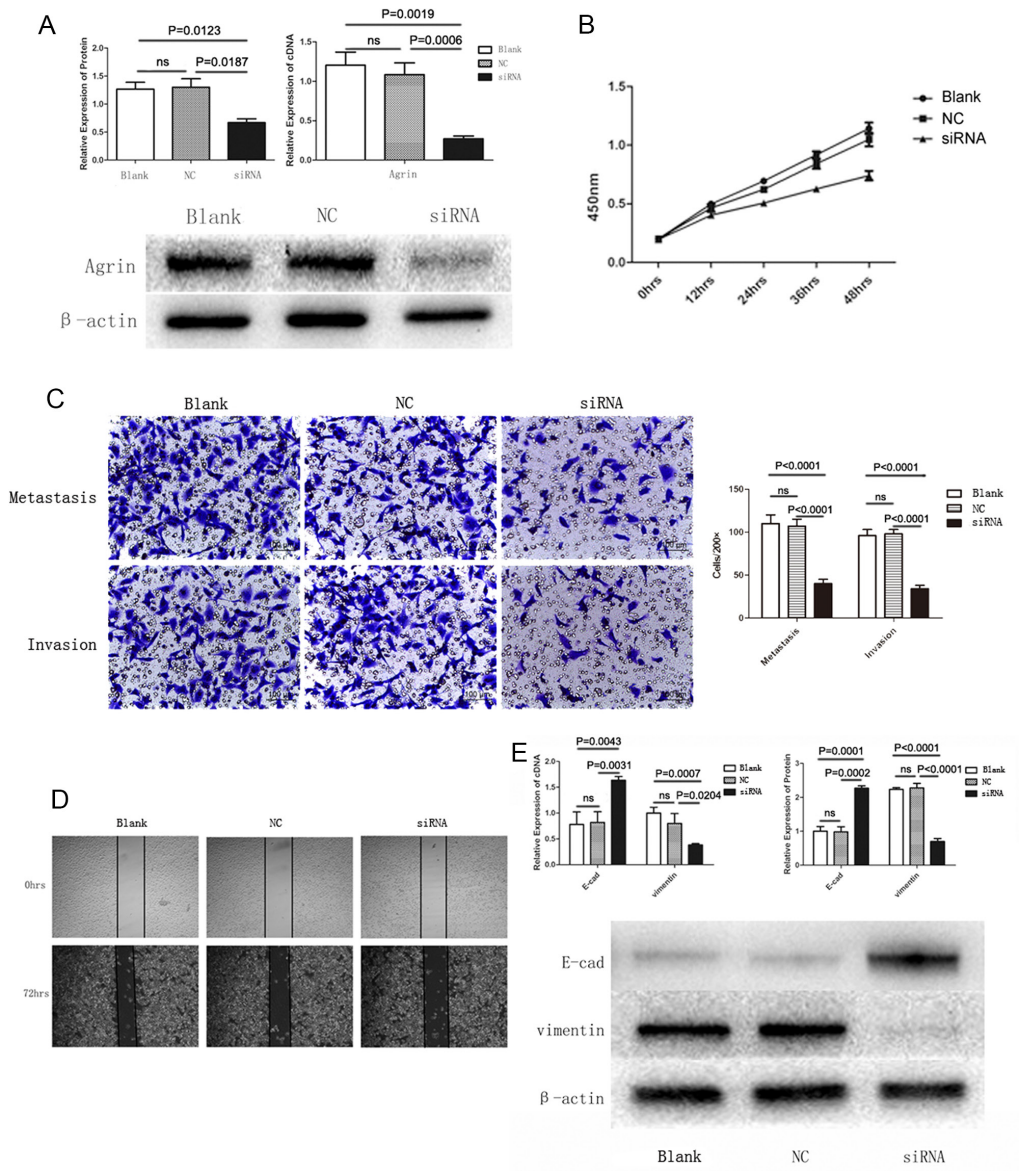
Agrin knockdown and its effect on cell proliferation, migration, and EMT **(A)** The effect of siRNA transfection on agrin expression. Both agrin protein and cDNA levels were downregulated in cells transfected with siRNA versus controls. **(B)** Absorbance curves showing cell proliferation ability of agrin knockdown (siRNA) cells versus controls. The agrin knockdown cells showed a markedly lower ability to proliferate. **(C and D)** Transwell migration (C) and wound healing (D) assays showing the metastatic and invasive ability of the cells. Both the invasive and metastatic abilities of agrin knockdown cells were impaired in comparison with the controls. **(E)** Western blot showing the reversal of the EMT process in agrin knockdown cells. E-cadherin was upregulated while vimentin was downregulated in the knockdown sample.

**Figure 6:**
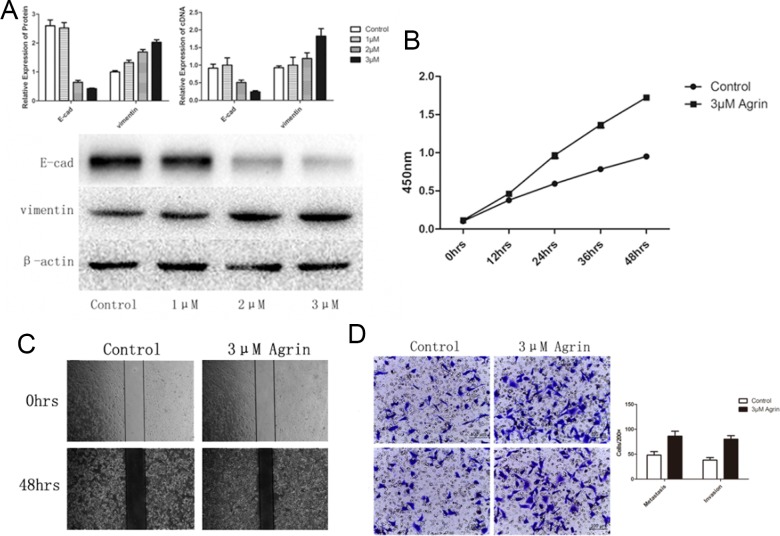
Effect of agrin supplementation on cell proliferation, migration, and EMT of SMMC-7721 cells **(A)** The effect of agrin supplementation on the expression of the EMT marker vimentin. Increasing concentrations of agrin caused an upregulation of vimentin expression and a lowering of E-cadherin expression. **(B)** Cells supplemented with 3-μm agrin showed a marked increase in their ability to proliferate. **(C and D)** Wound-healing (C) and transwell migration (D) assays showing the metastatic and invasive ability of the cells. Both the invasive and metastatic ability of the cells supplemented with 3-μm agrin increased in comparison with the controls.

To further characterize the functional role of agrin in HCC, we depleted agrin in the highly metastatic SMMC-7721 cells by transfecting with a short interfering RNA (siRNA) pool, by which effective agrin knockdown was achieved (Figure 5A). In the agrin knocked-down cells, cellular proliferation rates were reduced by 40% compared with the control (Figure 5B). Apart from this, agrin knockdown also achieved a significantly lower (>50% reduction) infiltration rates in transwell migration and invasion assays than control cells (Figure 5C). Furthermore, agrin depletion severely reduced the migration of SMMC-7721 cells in a wound-healing assay (Figure 5D). Furthermore, downregulation of agrin by siRNA could reduce the expression of vimentin and increase the expression of E-cadherin significantly, which is the typical phenomenon of EMT (Figure 5E). On the contrary, 3-μM soluble recombinant agrin significantly decreased the expression of E-cadherin and increased the expression of vimentin at both RNA and protein level in SMMC-7721 (Figure 6A). The same concentration of agrin could increase the proliferation, metastasis, and invasion ability of SMMC-7721 cells as proved by CCK-8 and trans well assays (Figure 6B and 6C and 6D).

**Figure 7:**
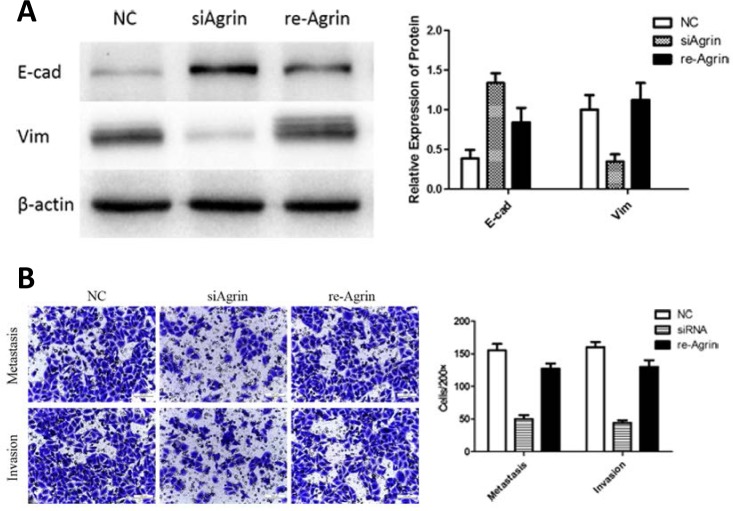
Effect of recombinant agrin on invasion and metastasis of SMMC-7721 cells **(A)** Treatment of siRNA transfected cells with recombinant agrin restored the expression of EMT marker vimentin. Transwell migration assay **(B)** showing the metastatic and invasive ability of cells upon treatment with recombinant agrin.

We next rationalized that restoring agrin should rescue the effects caused by agrin siRNA if these phenotypes are primarily attributable to reduced levels of agrin. Hence, we added 3-μM soluble recombinant agrin in agrin knockdown SMMC-7721 cells. The results indicated that recombinant agrin rescue the metastasis, and invasion ability of SMMC-7721 cells as proved by transwell assays (Figure 7B). Compared with the agrin-depleted group, agrin restoration increased vimentin by 2.9 folds and reduced E-cadherin by 48% (Figure 7A). These results cumulatively validate the role of agrin as a secreted sensor regulating E-cadherin and vimentin-dependent cell attachment.

### Sorafenib alleviates hepatocarcinogenesis, liver inflammation, and fibrosis *in vivo*

**Figure 8:**
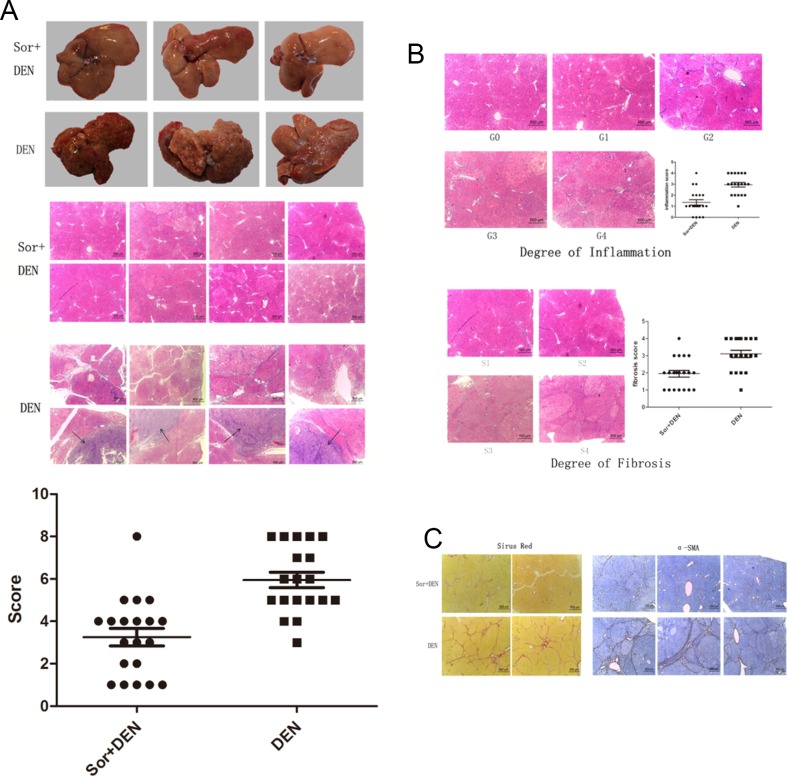
Effect of sorafinib on DEN-induced hepatocarcinogenesis in rats **(A)** Macroscopic hepatic changes in DEN and DEN + sorafenib-treated rats. Macro-nodular cirrhosis and several nodules were observed in the livers of DEN-treated rats, whereas DEN + sorafenib-treated liver specimens were almost normal. Liver sections from DEN-exposed rats showed abnormal architecture with hepatocellular necrosis, apoptosis, fibrous septum, hyperchromatic nuclei, and hepatocellular carcinoma, whereas sorafenib-treated rats mainly revealed normal architecture and histology with slight hepatic lesions. **(B)** The degree of inflammation and fibrosis was much lower in the sorafinib + DEN group compared with the DEN group. **(C)** Collagen and α-SMA decreased in sorafenib-treated rats.

At the 14^th^ week, rats of either group were sacrificed and their livers excised for examining the morphological and macroscopical hepatic changes. Several nodules were found scattered throughout the liver specimens of all DEN-treated animals resembling the texture of macro-nodular cirrhosis. On the contrary, the liver specimens of the rats preconditioned with sorafenib were almost normal. Histological liver lesions induced by DEN are summarized in Table 1. The liver sections from DEN-exposed rats showed an abnormal structure, with irregularly shaped cytoplasm containing hepatocellular necrosis and apoptosis, a large number of fibrous septum, enlarged and hyperchromatic nuclei, and even HCC. Other abnormal characteristics such as pseudo-nucleoli, pleomorphic nuclei, and irregular lipid droplets were also observed in the DEN-exposed group. Microscopic evaluation of liver sections from the sorafenib-treated rats exposed to DEN mostly revealed normal architecture and histology. However, slight hepatic lesions were also observed, including hepatocellular necrosis and fibrosis located in the portal area. HCC was observed in 1 liver (Figure 8A). However, the hepatocarcinogenesis rate was significantly lower in Group A than in Group B (*P*<.0001), considering if grade 6 and higher were defined as hepatocarcinogenetic (Figure 8A). Furthermore, liver sections also revealed inflammation and fibrosis at varying degrees as represented in Table 2 (Figure 8B) and sorafenib significantly improved portal inflammation (*P*<.0001) and fibrosis (*P* =.0003). The levels of both collagen and α-SMA were also significantly reduced in sorafenib-treated rats compared with the control (Figure 8C).

**Table 1:** Histological liver lesions induced by DEN

Grades	Pathological changes
0	No apparent lesions
1	Spotty necrosis scattering in hepatic lobule and inflammatory cell infiltrating
2	Steatosis of hepatocyte and localized necrosis
3	Pseudo-lobuli and tubercle, less than 50%
4	Pseudo-lobuli and tubercle, more than 50%
5	Mild atypical hyperplasia and suspicious oncogenesis
6	Moderate atypical hyperplasia and suspicious oncogenesis
7	Severe atypical hyperplasia and suspicious oncogenesis
8	Hepatocellular carcinoma

The grades from 0 to 8 represented a sequence of lesions with increasing severity.

**Table 2:** Degree of inflammation and fibrosis

Degree	Portal area and around	Interlobular change of liver
***Degree of inflammation activity***		
G0	No inflammation	No inflammation
G1	Portal area inflammation	Degeneration and little spotty necrosis
G2	Mild piecemeal necrosis or acidophilic body	Degeneration, spotty, or localized necrosis
G3	Moderate piecemeal necrosis	Degeneration and bridging necrosis
G4	Severe piecemeal necrosis	Extensive bridging necrosis involving multiple lobules
***Degree of fibrosis***		
S0	No fibrosis	
S1	Minimal portal fibrosis, localized in hepatic lobule	
S2	Fibrous septum formation and untouched structure of hepatic lobule	
S3	Fibrous septum formation and disorganized structure of lobule without cirrhosis	
S4	Early stage of cirrhosis	

During the experimental period and at the end of the assay, none of the rats showed external clinical signs of disease except for the death of a DEN-exposed sorafenib-untreated rat because of intestinal obstruction.

### Sorafenib improves liver function by regulating serum hepatic biochemical markers

**Figure 9:**
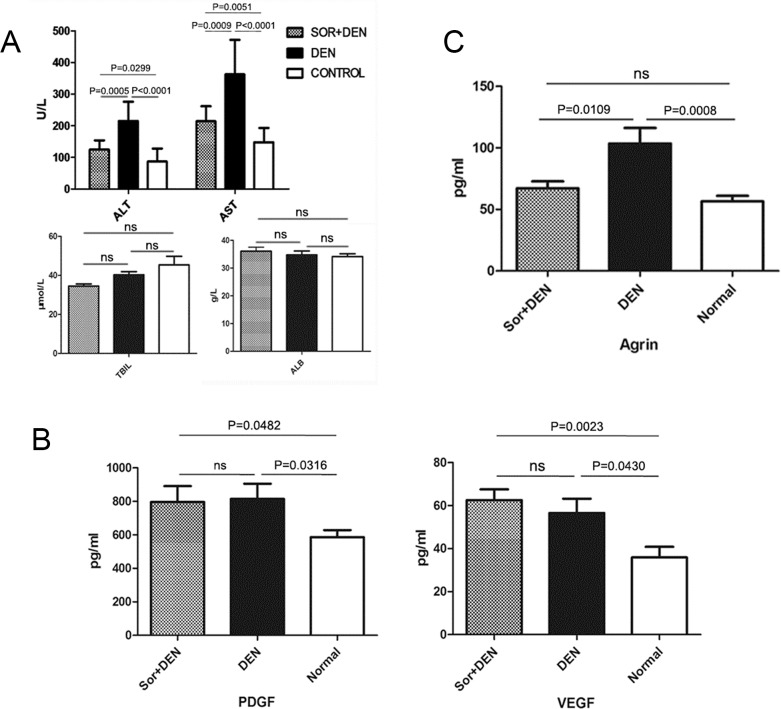
The effects of sorafenib on biochemical parameters in rats induced to develop HCC using DEN **(A)** Effects of sorafenib on liver enzyme levels, total bilirubin, and albumin. While sorafenib inhibited the upregulation of ALT and AST, no effect was observed on the levels of TBIL and ALB when compared with rats treated with DEN alone. **(B)** Effects of sorafenib on PDGF and VEGF levels. No effect was observed on the expression levels of PDGF and VEGF when compared with rats treated with DEN alone. **(C)** Effect of sorafenib on agrin levels. Treatment with sorafenib significantly reduced agrin levels when compared with rats treated with DEN alone.

A part of the liver homogenate was used for serum biochemical analysis of hepatic markers such as ALT, AST, ALB, and TBIL. ALT and AST levels were significantly lower in sorafenib-treated rats than in DEN-exposed rats. However, ALB and TBIL levels remained same with or without sorafenib compared with the negative control (Figure 9A). The serum levels of PDGF and VEGF were also measured and the results showed that the DEN-exposure could increase PDGF and VEGF significantly irrespective of the presence or absence of sorafenib treatment (Figure 9B). The agrin serum level of sorafenib-treated rats was significantly lower than the only DEN-exposed rats (Figure 9C).

## DISCUSSION

Liver fibrosis is the main pilot process of HCC that results from chronic inflammatory damage to the liver in conjunction with the accumulation of ECM, which is secreted by the activated HSC [7, 16]. Therefore, the activation of HSC may play an important role in the process of hepatocarcinogenesis [17–19]. Cellular and molecular experiments of our study demonstrated that PDGF is an effective activator of HSC, which was proved by the increase of α-SMA and collagen I, markers to predict activation. Sorafenib could inhibit the HSC activation by blocking the combination of PDGF and PDGFR. The cellular immunofluorescence analysis proved the same function of PDGF. As an important regulator, ERK phosphorylation may lead to numerous cellular variations. Our results showed that the mechanism of HSC activation by PDGF is probably regulated by the increased phosphorylation of ERK.

HSCs exhibit biological functions that influence the onset and the progression of HCC. Thus, HSCs can induce phenotypic changes in hepatocytes notably through the production of growth factors and cytokines (eg, MMP, PDGF, TGF-β, FGF, and VEGF) in favor of tumor cell proliferation [20]. In our study, co-culture with activated HSC could increase the proliferation, migration, and invasion ability of normal hepatocyte cell line and HCC cell line. EMT was also induced by this approach. E-cadherin, the marker of epithelial cells, decreased and vimentin, which represents mesenchymal cells, increased, indicating EMT, which indicates a decrease in cell-to-cell adhesion and enhancement of cell migration. Studies indicate that expression of EMT markers E-cadherin and vimentin could be used as prognostic markers of metastasis in HCC [21]. This phenotypic change of EMT was observed in our study that is the key procedure of oncogenesis and cancer metastasis. Therefore, the results demonstrated that PDGF could activate HSCs, which promoted the proliferation, migration, and invasion as well as EMT of normal and cancer cell lines that may induce the progress of hepatocarcinogenesis.

We studied the differences between the supernatant of activated HSCs and quiescent HSCs to observe enriched proteins expression and were able to characterize agrin expressed either as membrane protein or secreted in ECM. We further elucidated the mechanistic role of agrin by agrin knockdown using siRNA, which decreased the proliferation, metastasis, and invasion ability of HCC cell line and promoted MET (Figure 5C and 5E). The study by Chakraborthy et al showed reversal of EMT characteristics and loss of invasiveness following agrin knockdown, suggestive of its role in oncogenesis. Conversely, exogenous addition of soluble agrin increased migration, invasion, and mesenchymal characteristics [12]. Studies have shown accumulation of agrin in cirrhosis and HCC both in humans and rats [13]. Our results show that agrin plays the similar role as the supernatant of activated HSCs to the HCC cell line, indicating that agrin is the main contributing factor in the activated HSC supernatant, suggesting its role to promote the process of hepatocarcinogenesis and HCC development by proving oncogenic microenvironment. Though it remains unclear as to how PDGF binding to its receptor stimulates agrin secretion in HSCs. The plausible mechanism by which agrin regulates cell-proliferation, invasion, migration and EMT is through Lipoprotein-related receptor-4-muscle-specific receptor tyrosine kinase) signaling [12].

The process of transformation from chronic liver injury to liver fibrosis and to HCC has several chronic inflammatory events that trigger the molecular and cellular mechanisms leading to transformation [22]. To understand this mechanism, in our study, we selected a well-known model of hepatocarcinogenesis induced by DEN to SD rats to mimic pathophysiological features of liver disease leading to HCC development in humans where a combination of etiological factors such as inflammation injury and advanced fibrosis and cirrhosis are most likely to play a role. Sorafenib was administrated as a protective measure to know the mechanism of its action at various stages of development of HCC. Studies have reported the use of tyrosine kinase inhibitors in treatment of cirrhosis, eventhough it is not the standard treatment recommended by guidelines, sorafinib has reported to reduce the degree of cirrhosis. Similar results were obsereved in pre-clinical study using sunitinb. Our results showed that sorafenib contributed to the prevention of DEN-induced hepatocarcinogenesis in rats and could also alleviate the liver inflammation and fibrosis degree during the carcinogenesis process. This indicates the protective effect of sorafenib at the early stage of oncogenesis and also indirectly the role of PDGF in oncogenesis. It is observed that inflammatory damage to the liver starts the HSC activation to secrete collagen and activated HSCs secrete inflammatory chemokines and growth factors, which modulate the activation of HSC. Therefore, a vicious circle in which fibrogenesis and HSC stimulate each other is most likely to occur. Our results suggested that sorafenib could protect hepatocytes during fibrogenesis by breaking the circle to prevent fibrogenesis. Overall, our results suggest that sorafenib could be a potential drug in pathological conditions like cirrhosis.

The targets of sorafenib are PDGFR and VEGFR. It is still unclear if the serum levels of PDGF and VEGF would be influenced by sorafenib. To elucidate this, we detected the serum levels of PDGF and VEGF. The results indicated that DEN increased PDGF and VEGF, which in turn contribute to hepatocarcinogenesis. It is observed that sorafenib could not decrease the levels of PDGF and VEGF, but it alleviated the inflammation, fibrosis, and rate of oncogenesis by blocking the connection of PDGF and PDGFR.

On contrary, the decreased ALT and AST levels in sorafenib-treated rats indicate the protection to hepatocyte damage, because both of these increase in hepatocyte injury. The ALB level remained unchanged between sorafenib-treated and untreated rats, partly demonstrating that the DEN-induced oncogenesis does not destroy the synthetic ability of hepatocytes. Moreover, agrin level of sorafenib-treated rats was significantly lower than the sorafenib-untreated rats, implicating DEN-induced liver inflammation by activated HSC through agrin secretion. On the other hand, sorafenib inhibited the activation of HSCs, reduced agrin secretion, thereby alleviating fibrosis and HCC, demonstrating the oncogenic role of agrin and protective role of sorafenib in hepatocarcinogenesis.

The strength of this study lies in the rationale that this is the first time the role of agrin in HCC with regard to the PDGF-HSC mitogenic pathway has been assessed and has been shown the mechanism underlying the oncogenic role of agrin. However, the nature of the study being a pre-clinical study, which projects the findings on laboratory- and animal-based outcomes may be a limitation. However, the findings may be preliminary and may form the basis of future clinical studies to elucidate the mechanistic approach of oncogenic property exerted by agrin in humans. We suggest that, agrin and PDGF may prove to be the crucial targets for the treatment of liver cirrhosis and HCC, and sorafenib is one such targeted drug that has been already approved for HCC.

In summary, PDGF can induce HSC activation, which could be inhibited by sorafenib. The main mechanism of the inhibiting the activation of HSC was by inhibition of the combination of PDGF and PDGFR and downregulation of the phosphorylation of ERK. Paracrine of agrin by activated HSC can induce the proliferation, metastasis, invasion, and EMT of HCC cell lines. Sorafenib could inhibit the hepatocarcinogenesis both *in vivo* and *in vitro*. This may provide a new possible method for the prevention of HCC.

## MATERIALS AND METHODS

### Supernatant collection and protein enrichment

To investigate the differential expression of proteins between the supernatant of quiescent and activated HSC, LX-2 cells were cultured in two culture bottles with same cell count with serum-free medium to keep LX-2 quiescent. The supernatant was replaced with an equal amount of fresh serum-free medium in 2 days, and 20 ng/mL of PDGF (with or without sorafenib) was added into one bottle for another 24 hours. The supernatant and the cellular proteins of the 2 bottles were collected. The activation of the PDGF-treated LX-2 cells was measured in terms of increased α-smooth muscle actin (SMA) detection. Furthermore, the supernatant of activated LX-2 cells was used to culture HL-7702 or SMMC-7721 cells by mixing with 10% FBS to access the proliferation and wound-healing capability. For protein isolation, the culture medium was centrifuged at a high speed at 4°C and 1-mL supernatant was transferred into a new 1.5-mL Eppendorf. The proteins were enriched by lyophilization.

### Liquid chromatography and mass spectrometry analysis

Proteins in the conditional supernatant were extracted and enriched as described above, and the powder in the Eppendorf tubes were re-dissolved in 10-μL tri-distilled water and separated on a 10% SDS-PAGE. The extracted peptides were subjected to liquid chromatography analysis to see the differences in proteins expressed in quiescent and activated LX-2 cells. The metabolite signals were obtained following baseline correction and peak alignment. T-score method was adopted to identify the featured peaks differing between supernatant of activated and quiescent LX-2 cells. Equal amounts of re-dissolved powder were separated on a one-dimensional gradient Bis-Tris gel and digested with trypsin. The samples were analyzed on full-scan mass spectra. Identification and quantification of peptides were performed using mascot version 2.

### Co-culture of LX-2 and HL-7702 or SMMC-7721

HL-7702 or SMMC-7721 (1 × 10^5^ cells/well), in serum-free media were seeded in the upper trans well chambers and immortal LX-2 cells in the lower insert. During a 48-hour incubation period at 37°C, 5% CO_2_, the media, was replenished every 24 hours and the co-culturing was continued for 4 days. The insert was fixed in ethanol and stained with crystal violet. The number of the cells on the lower surface was counted by microscope. Following this, RNA and cellular protein of HL-7702 or SMMC-7721 were extracted and detected. The invasion assay was performed in the same manner as the migration assay except that the upper chamber was precoated with 50 g/well of Matrigel overnight before seeding the cells.

### RNA preparation, reverse transcription, and quantitative real-time PCR

Total RNA was extracted from conditional cultured cells using Trizol. M-MLV Transcriptase (Takara) was used for reverse transcription. Quantitative real-time PCR was performed using Premix Ex Taq (Takara). Primers used for specific genes are represented in Table 3. The expression levels of target genes E-cadherin and vimentin were normalized to the housekeeping gene *GAPDH*.

**Table 3:** Primers of target genes

Gene	Primers (5′—3′)	
E-cadherin	Forward primer	ATTTTTCCCTCGACACCCGAT
	Reverse primer	TCCCAGGCGTAGACCAAGA
Vimentin	Forward primer	AGTCCACTGAGTACCGGAGAC
	Reverse primer	CATTTCACGCATCTGGCGTTC
Agrin	Forward primer	CCGCCAGGAGAATGTCTTCAA
	Reverse primer	TTTCGTAGGTGACTCCGTCGT
GAPDH	Forward primer	GAGCCACATCGCTCAGAC
	Reverse primer	CTTCTCATGGTTCACACCC

### Western blot analysis

The conditional cultured cells were homogenized in extraction buffer and centrifuged at 14,000 g for 20 min to collect the lysate. Eighty micrograms of total protein was separated by SDS-PAGE electrophoresis and then transferred to PVDF Transfer Membranes, which were blocked with 5% nonfat milk dissolved in washing buffer, probed with appropriate primary antibodies against target proteins, and detected by anti-rabbit or anti-mouse secondary antibodies. The signal intensity was analyzed by Image J software. For phosphor-proteins, bands were normalized to their respective total protein levels, whereas for other panels, bands were normalized to a loading control (β-actin)..

### Wound-healing assay

Cell migration was assessed by wound-healing assays. Confluent SMMC-7721 cells were plated on six-well plates and wounded by manual scratching with a 200-μL pipette tip. At the specific time points, phase contrast images at specific wound sites were taken.

### Cell proliferation assays

Cells were seeded in 96-well plates, and cell proliferation was measured at 0, 12, 24, 36, and 48 hours by Cell Counting Kit 8 assay according to the manufacturer's instructions. Each experiment included 6 replicates and was repeated 3 times.

### Trans well migration and invasion assays

Cell invasion was determined using the 24-well chambers with 8-mm pore poly carbonate membranes either uncoated (for migration) or precoated with Matrigel Basement Membrane Matrix (for invasion). The chambers were rehydrated in serum-free medium. Complete medium with 700-μl 10% FBS served as chemo-attractant in the bottom chamber and 1 × 10^4^ cells per milliliter cells were incubated for 24 hours at 37°C, 5% CO_2_. At the end of the incubation period, cells invading membrane or Matrigel were washed and stained with 0.05% crystal violet solution and imaged in a bright-field microscope.

### Immunofluorescence

Cells cultured on the slides were washed 3 times with PBS and fixed with 4% paraformaldehyde at 4°C for 30 min. After another wash, the cells were permeabilized in PBS with 0.1% Triton X-100 for 15 min at room temperature and then blocked with 10% donkey serum albumin. Cells were incubated with primary antibodies overnight at 4°C, then with fluorescence labeling using secondary antibody for 1 hour at room temperature followed by incubation with DAPI for 10 min. The slides were mounted with an anti-fluorescence quenching agent and observed under an Olympus fluorescence microscope.

### *In vivo* hepatocarcinogenesis

**Figure 10:**
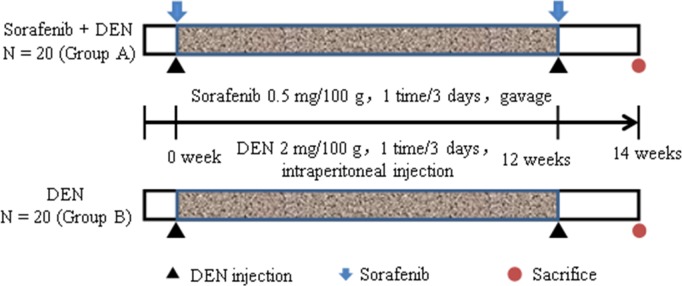
Experimental design Experimental SD rats were divided into 2 groups of 20 rats each. Injections of DEN (Group B) and DEN with sorafenib (Group A) were carried out as indicated. All the rats were sacrificed at 22 weeks of age after which the tissues were obtained for immunohistological analysis.

A total of 40 Sprague Dawley rats were purchased from the Experimental Animal Center, Chinese Forth Military Medical University, and were divided into 2 groups. Group A received DEN and sorafenib (10 μM, intragastric administration) and Group B received diethylnitrosamine (DEN) for HCC induction. The study protocol was approved by the Institutional Ethics Committee of Xijing Hospital. To address the influence of sorafenib on rats with DEN-induced hepatocarcinogenesis, the rats of both groups were intraperitoneally injected with DEN every 3 days for 12 weeks, starting at 8 weeks of age. Sorafenib was administered intragastrically to the rats in Group A every DEN injection. The rats were sacrificed at 22 weeks of age (Figure 10).

### Immunohistological analysis

Liver tissues were fixed with 10% formalin, embedded in paraffin, and cut into 4-μm sections for staining with hematoxylin-eosin (H&E). For quantitative assessment of fibrosis, sections were stained with Sirius red for collagen content. To define the activation of HSCs, levels of α-SMA were assessed by staining with rabbit anti-α-SMA antibody and visualized with HRP-labeled anti-rabbit antibody.

### Serum biochemical analysis

The blood of rats was collected in anti-coagulative tube and centrifuged at 1500 rpm for 20 min after standing for 30 min at room temperature. The serum was added into the serum automatic biochemistry analyzer to detect ALT, AST, ALB, and TBIL. The growth factors PDGF and VEGF as well as the secreted protein agrin were detected using ELISA kits.

### Statistical analysis

All continuous variables were presented as mean ± standard deviation (SD). For *in vitro* experiments, bar charts represent mean values, and error bar indicates SD. For *in vivo* experiments, error bars represent SEM. A paired “two-tailed” Student *t*-test and chi-square tests were performed using GraphPad Prism software, and data were considered significant at *P* <.05.
